# Unrecognized human immunodeficiency virus infection and risk factors among elderly medical patients at the Korle Bu teaching hospital, Accra, Ghana

**DOI:** 10.1186/s40794-016-0034-9

**Published:** 2016-09-02

**Authors:** Andrew A. Adjei, Seth Agyemang, Francis D. Krampa, Mubarak Abdul-Rahman, Francis Ofei, Margaret Lartey, Theophilus K. Adiku, Richard K. Gyasi, Yao Tettey

**Affiliations:** 1 Office of Research, Innovation and Development, University of Ghana, Legon, Accra, Ghana; 2 Immunology Department, Central Laboratories, Korle Bu Teaching Hospital, Accra, Ghana; 3 Department of Microbiology, School of Biomedical and Allied Health Sciences, College of Health Sciences, University of Ghana, Accra, Ghana; 4 Department of Pathology, School of Biomedical and Allied Health Sciences, College of Health Sciences, University of Ghana, Accra, Ghana; 5 Department of Medicine and Therapeutics, University of Ghana School of Medicine and Dentistry, Accra, Ghana

**Keywords:** HIV, Hospital, Admission, Elderly, Ghana

## Abstract

**Background:**

The human immunodeficiency virus (HIV) infection usually infects persons in the reproductive age group (15–49 years), but elderly people are also susceptible. Many people in sub-Saharan Africa including Ghana believe that elderly people are not at risk for HIV. Despite numerous reports of the high prevalence of HIV infection among the elderly worldwide, there are no from Ghana. This work determined the sero-prevalence of HIV infection and risk factors for its transmission among 1,100 hospitalized elderly people at the Korle Bu Teaching Hospital (KBTH), Accra, Ghana.

**Methods:**

Subjects voluntarily completed a risk-factor questionnaire and provided a blood specimen for HIV testing.

**Results:**

Of the study participants, 440 were male (mean age: 64 ± 10.55 years), and 660 were female (mean age: 63 ± 9.51 years). The overall HIV-1 sero-prevalence among the subjects was 4.18 % (*n* = 46). On multivariate analysis, there was no statistical significance between the socio-demographics or risk factors and the HIV status of the participants.

**Conclusion:**

The results suggest high prevalence of HIV-1 among hospitalized elderly people at KBTH, recommending the need to include the elderly in HIV/AIDS testing, prevention, and control programmes.

**Trial registration:**

Trial registration number: MS-Et/M.9 - p4.10/2012–2013. Registered: 10th April, 2013.

## Background

Human immunodeficiency virus (HIV) infection exists worldwide with particularly high rates of infection in sub-Saharan Africa [1–3]. Of the 1.8 million HIV/AIDS related-deaths recorded worldwide in 2010, 1.2 million were from sub-Saharan Africa [4]. The burden of HIV/AIDS disease is higher in young people (15–49 years old) than in older people (>50 years old) [4, 5] and as a result, most of the known epidemiological and clinical features of HIV/AIDS were defined and continued to be defined using younger populations [4–7]. Subsequently, most of the HIV/AIDS prevention efforts largely target young persons and people in reproductive age group. Little is known about the attitudes towards HIV and awareness of prevention, testing and treatment among older people in sub-Saharan Africa.

Ghana, one of the growing economies in sub-Saharan Africa, has been experiencing the HIV/AIDS epidemic since the first Ghanaian tested positive for HIV-1 antibodies in 1986 [8]. The prevalence of HIV infection in Ghana has rapidly decreased over the past years and the current prevalence rate is 1.37 % [9]. Sentinel studies conducted by the National AIDS control Programme (NACP) included most sub-populations thought to be at high-risk for HIV and sexually transmitted infections (STIs), but excluded older people (>50 years old) [9]. Recent reports indicate that the diagnosis of HIV infection is occurring with increasing frequency in older people [10–12]. The Centers for Disease Control and Prevention (CDC) reported that HIV/AIDS cases among American adults over 50 years of age quintupled during the last decade [7]. Similar results were reported in France [13], Australia, Canada, United Kingdom [14], Brazil [13], Egypt [15], Tanzania [16], Kenya [17], and Uganda [10, 14].

Anecdotal reports from the Department of Medicine and Therapeutics, Korle Bu Teaching Hospital (KBTH), Accra, Ghana, indicate a rise of older people (>50 years old) on admission at both the Surgical/Medical Emergency (SME) Ward and the Medical Wards, Department of Medicine and Therapeutics, KBTH; and that, age- related illnesses were the leading cause of death among the elderly (>60 years old) on admission. Although several studies have indicated that older patients with HIV infection present with non-specific problems or signs and symptoms that mimic age-related illnesses [18], the testing and diagnosis of HIV-related immune-suppression were not done, unrecognized or overlooked among the patients on admission in both wards. Consequently, the sero-prevalence of HIV and/or HIV/AIDS-related death among the elderly patients was not included in the report.

Several reports are available globally on the sero-prevalence of HIV among the elderly patients on admission in hospitals [15, 16, 19, 20] and also in the general population [17, 21–23]. However, in Ghana, HIV infection among older people has largely been ignored over the years with little or no information on the sero-prevalence and HIV-related knowledge and attitudes among older people. Some of the factors that may explain the lack of interest or late diagnosis of HIV infection in the older people may include less common routine screening, lack of appreciation of physicians and/or clinicians of HIV infections in the elderly, and the lack of information on the contribution of HIV infection to the morbidity in older people. Additionally, there is confusion between symptoms of opportunistic infections and those of frequent co-morbid conditions associated with ageing. In Ghana, most HIV/AIDS health policies and programmes target the traditionally vulnerable (substance users, patients seen at STI clinics and incarcerated persons), women and children, without any focus on elderly populations. Similarly, research studies typically exclude older participants [9]. This work therefore determined the sero-prevalence of HIV infection among hospitalized elderly patients at KBTH. We also examined the association of HIV with various suggested risk factors for its transmission.

## Methods

### Study site description

The study is a hospital-based, cross sectional study carried out between the months of December 2012 to January 2014 among elderly patients on admission at the Surgical Medical Emergency (SME) and Medical Wards at the KBTH. Ghana is divided into ten (10) administrative regions, subdivided into a total of 216 districts or municipalities. KBTH, situated in Accra is the leading tertiary hospital and the major referral centre in Ghana. It also serves as the teaching hospital of the University of Ghana School of Medicine and Dentistry (UGSMD), Accra, Ghana. Both the KBTH and the UGSMD are situated in the Greater Accra Region. The SME/Medical Wards are its biggest tertiary care centres and more than 40 % of the total cases involving older people seen in the country are processed through the Department of Medicine and Therapeutics.

### Study population

Subjects for this study included both male and female patients aged 50 years and older admitted in both SME and the Medical Wards. Patients originated from various social and ethnic groups as well as geographically distinct areas from the vast territory of all the 10 regions of Ghana. Patients who were not admitted to these wards, whose HIV/AIDS status were known or were critically ill were not included in the study. The study was presented to the entire population of each ward at dedicated research meetings. The patients were informed that the study was confidential and that the information provided would not affect their admission or treatment status. The objective of the study was clearly explained to all potential participants in any local Ghanaian language that they best understood, after which a written informed consent was obtained and kept at the principal investigator’s office. A total of 1,100 patients consented to participate in the study.

### Questionnaire

All 1,100 consenting patients completed an interview-based questionnaire assessing socio-demographic characteristics, including age, sexual orientation, drug and alcohol histories, knowledge and history of HIV/AIDS, STIs, and a risk factor profile for the infection under investigation. The interviews were conducted by trained nurses.

### Sample collection and serological analysis

Blood samples (approximately 5 ml) were collected from each of the 1,100 participants into EDTA tubes. Samples were centrifuged (2500 rpm for 10 min) and the separated plasma kept at −80 °C until analysed. Plasma were screened for HIV (1 & 2) antibodies/antigens with a COBAS e411 immunoassay (ROCHE Diagnostics, Manheim, Germany). Reactive specimens were re-tested with reverse transcription polymerase chain reaction, RT-PCR (ROCHE Diagnostics) at the Immunology and Cell Biology Department, Central Laboratory, KBTH, in accordance with the respective manufacturer’s instructions.

### Data analysis

The Statistical Analysis Software (SAS Institute, Cary, NC, USA) version 9.1 was used to complete all data analyses. For each generally accepted risk factor for HIV infection, the odds ratio (OR) and the 95 % confidence interval (95 % CI) were calculated to assess associations with socio-demographic and behavioural variables in univariate analysis. A *P*-value of <0.05 was considered significant and the Fisher’s exact test was used in estimating significance in relatively small frequencies. Independent associations were evaluated by calculating the adjusted OR using multivariate analysis for the socio-demographic variables found to be significant in the univariate analysis.

## Ethical approval

The Ethical and Protocol Review Committee of the University of Ghana, College of Health Sciences approved the study prior to enrolment.

## Results

### Patient distribution, age and clinical conditions

A total of 1,100 people participated in the study. Of the participants, 440 (40 %) were male (mean age: 64 ± 10.55 years), and 660 (60 %) were female (mean age: 63 ± 9.51 years). The participation for the study at the two sites ranged from 39.5 % (SME) to 60.5 % (Medical Block). All 1,100 participants completed interviews and blood testing. All the consenting elderly people were Ghanaians and data from male and female elderly people were combined.

Of the 1,100 patients, 75.18 % (827 out of 1,100) had been admitted at the hospital for less than 4 weeks while 24.82 % (273 out of 1,100) had been in hospital for more than 4 weeks. The most common cause for their admission was hypertension (blood pressure ≥ 140/90 mmHg) in 51 % (*n* = 561), diabetes in 37.55 % (*n* = 413) followed by cancer 5.64 % (*n* = 62). Other diagnoses accounted for 5.18 % of the study population. Eighty percent of the participants had never been tested for HIV.

### Prevalence of HIV among study patients

Forty-six (4.18 %) participants tested sero-positive for HIV-1 after COBAS e411 immunoassay and RT-PCR. None tested positive for HIV-2. The sero-prevalence of HIV-1 was highest in participants aged >75 years and lowest among participants aged 61–75 years (Table 1a).

**Table 1:** ORs and the corresponding 95 % CIs of age and gender for HIV sero-positivity among the study patients

	N [1,100]	HIV Status		OR	95 % CI	*p*-value
		Pos	Neg			
		(46)	(1054)			
Age						
50–60	498	24	474	^a^	-	-
61–75	459	11	448	0.49	0.24–1.00	0.03
> 75	143	11	132	1.65	0.79–3.45	0.13
Gender						
Male	440	19	421	1.06	0.5–1.93	0.48
Female	660	27	633	^a^	-	-

^a^: references for calculations of OR

Table 1 shows the ORs and corresponding 95 % CIs according to age, gender and HIV sero-positivity. Of the participants who tested positive for HIV-1, there was no significant difference by gender; 58.70 % (*n* = 27 out of 46) were female, while 41.30 % (*n* = 19 out of 46) were male. The younger group (ages 50–60) had higher rates of HIV sero-positivity (52.17 %; *n* = 24 out of 46) compared to elderly people aged 61–75 years (23.91 %; *n* = 11 out of 46) and those aged ≥ 76 years (23.91 %; *n* = 11 out of 46)) but the differences were not statistically significant.

Table 2 shows ORs and the corresponding 95 % CIs for demographic characteristics and HIV sero-positivity among the elderly people on admission. Married elderly people accounting for 86.91 % of the study participants had higher proportions of HIV sero-positivity compared with those who w not married. There were no statistically significant differences in demographics between the positives and negatives

**Table 2:** ORs and the corresponding 95 % CIs demographic factors and HIV sero-positivity among the study patients

	*N* [1,100]	HIV status		OR	95 % CI	*p*-value
		Pos (46)	Neg (1054)			
Education						
Illiterate	466	14	452	0.62	0.20–1.93	0.29
Basic	230	12	218	1.10	0.35–3.51	0.57
Secondary	320	16	304	1.05	0.34–3.24	0.60
Tertiary	84	4	80	^a^		
Marital Status						
Married	956	43	913	2.21	0.68–7.23	0.13
Unmarried	144	3	141	^a^		
Religion						
Christian	668	24	644	0.46	0.19–1.11	0.08
Muslim	338	15	323	0.58	0.23–1.46	0.18
Other	94	7	87	^a^		
Occupation						
Formal	231	8	223	1.47	0.47–4.57	0.35
Informal	659	33	626	2.16	0.83–5.61	0.07
Unemployed	210	5	205	^a^		

^a^: references for calculations of OR

Table 3 shows the ORs and corresponding 95 % CI according to behavioural characteristics and HIV sero-positivity. There were no statistically significant differences in behavioural characteristics between the positives and negatives.

**Table 3:** ORs and corresponding 95 % CI of HIV status according to behavioural character of the elderly hospitalized patients

	*N* [1,100]	HIV Status		OR	95 % CI	*p*-value
		Pos (46)	Neg (1054)			
Condom use						
No	1053	43	1010	0.67	0.20–2.23	0.35
Yes	47	3	44	^a^		
Concurrent sex partners						
No	702	27	674	^a^		
Yes	398	19	379	1.25	0.69–2.28	0.28
Drug use						
No	1088	46	1042	^a^		
Yes	12	0	12		-	0.60
Alcohol use						
No	663	28	635	^a^		
Yes	437	18	419	0.97	0.53–1.78	0.53
Smoking history						
No	979	42	937	^a^		
Yes	121	4	117	0.76	0.27–2.17	0.42
Chewing tobacco						
No	986	39	947	^a^		
Yes	114	7	107	1.59	0.69–3.64	0.19
History of STI						
No	1095	46	1049	^a^		
Yes	5	0	5	-	-	0.81
Symptoms of STI						
No	167	6	161	^a^		
Yes	933	40	893	1.20	0.50–2.88	0.44
Payment for sex						
No	985	40	945	^a^		
Yes	115	6	109	1.30	0.54–3.14	0.35
Tattoos						
No	633	28	605	^a^		
Yes	467	18	449	0.87	0.47–1.59	0.38
Blood transfusion						
No	568	27	541	^a^		
Yes	532	19	513	0.74	0.41–1.35	0.20

^a^: references for calculations of OR

Table 4 shows HIV awareness and knowledge in prevention and transmission among the study participants. The majority of respondents had heard of HIV and were aware of STIs. Only 26.10 % of the participants knew that correct and consistent use of condom could protect people from getting HIV, while 60.9 % of participants did not know that a person can protect himself/herself through abstinence. Seventy-three percent of participants thought that healthy-looking individuals could not be infected with HIV.

**Table 4:** HIV awareness among patients

HIV awareness	Response (%)	
	Yes	No
Have you heard of HIV/AIDS?	93.7	6.3
Are you aware of STIs?	87.7	12.3
Can correct use of condoms prevent HIV?	26.1	73.9
Can HIV be acquired through food?	25.8	74.2
Can HIV be acquired through handshakes/touch?	94.3	5.7
Can HIV be acquired through contaminated needles?	66.8	33.2
Can HIV be acquired through contaminated blood?	73.5	26.5
Can a person protect himself from HIV by abstaining?	39.1	60.9
Can healthy individual be infected with HIV?	37.0	63.0
Is there Medication to cure HIV?	42.3	57.7
Can pregnant woman pass HIV to unborn child?	21.4	78.6

## Discussion

To our knowledge, there are no studies that have assessed the prevalence rates of HIV and its impact on the health and well-being of the elderly people in the Ghanaian setting despite the increasing growth of the elderly population and the significant role(s) they play in both social and family settings.

Undocumented reports indicating a rise of older people (>60 years old) on admission at both the SME Ward and the Medical Wards, Department of Medicine and Therapeutics, KBTH among the elderly raises a major public health concern in the era of global HIV/AIDS pandemic. Notably, HIV/AIDS was not included in the cause(s) of death among the elderly emphasizing the perception that the elderly are at a little or no risk of HIV infection.

Our results of 4.18 % sero-prevalence rate among the elderly people on admission at the Wards suggest that HIV may pose a significant problem and that the detection and diagnosis of HIV among elderly people may remain unrecognized during their lives. The reason(s) for the failure of “early” detection of HIV among the elderly cannot be discerned from our study. However, one of the reasons may be the misconception of the association of the elderly with HIV. In the Ghanaian traditional society, the elderly are held in high esteem because of the leading and significant roles they play as heads of families, “fountain(s)” of wisdom, and roles in conflict resolutions. As such, many Ghanaians mistakenly believe that elderly people are not at risk and do not engage in risky behaviours. Another reason may be the failure of clinicians and/or physicians to document factors, such as homosexuality, previous history of drug use, condom use, and multiple sexual partners that are associated with HIV in elderly people. Although only three study participants identified as homosexual, two of them tested positive for HIV-1, suggesting that known HIV risk factors are also relevant in an elderly population. In addition to just not considering HIV in older patients, it is also possible that clinicians attribute symptoms of opportunistic infections to common aging-associated co-morbid conditions. Further studies are needed to better define the sero-prevalence of HIV in Ghanaians aged 50 and older.

The participants in this study demonstrated a significant lack of knowledge about HIV/AIDS and STI acquisition and transmission as well as ignorance of their own HIV status and risk. These findings should prompt further efforts to educate older Ghanaians about sexual health and to increase awareness among health care providers that HIV/AIDS in the elderly population is a salient and underappreciated issue.

The overall sero-prevalence of HIV among the elderly patients on admission at KBTH (4.18 %) was higher than the sero-prevalence in the general population (1.37 %) and that of similar studies in Cameroon (2.6 %) [12]. The result is comparable to studies conducted in north-western Ethiopia (5 %) [21], but lower than the reported sero-prevalence of HIV in elderly individuals in Tanzania (15 %) [20]. The sero-prevalence of HIV infection among the elderly people admitted at both SME/Medical Wards, KBTH suggests that HIV is likely prevalent in the elderly inpatient populations in other districts. Further studies to be done among the elderly inpatient populations in other health facilities in Ghana to measure the sero-prevalence and define the risk factors associated with HIV infection in this valued group.

The risk of HIV did not correlate with increasing age, however, elderly people aged > 75 years on admission tended to have a higher proportion of HIV infection (Table 1). Similar findings were noted in a study conducted by Mtei & Pallangyo, 2001 in Muhimbili Medical Center, Dar es Salaam, Tanzania. The reason(s) for this disparity cannot be discerned from this study and hence, there is the need for further studies to be done to define the impact of HIV on the health and well-being of elderly people in the population.

Another finding of interest reported herein in this study is that a greater number of sero-positive participants (*n* = 33 out of 46) were in the informal employment sector. They consisted mainly traders who travelled long distances for several weeks to engage in trading activities. A higher HIV sero-positivity in traders may be attributed to separation from partners and family, peer influence, alcohol and drug use, low perceived vulnerability to HIV infection and freedom from social norms. The higher sero-prevalence among this group, especially traders who travel long distances and spend several weeks away from home is suggests that the traders may constitute a bridge or core population in the spread of HIV and other STIs which would be consistent with existing literature [24–26]. This finding presents another opportunity for further research to investigate the sero-prevalence of HIV in elderly traders and any correlation with geographic spread of the infection.

As documented in other studies, condom use was rare in this population of elderly inpatients [27–29]. Of the 46 participants who tested positive for HIV, 43 reported no history of condom use during sexual intercourse.

There are many limitations to this study. The small sample size, inability to collect information on sexual practices prior to admission, likely under-reporting of sexual activity, alcohol use and other risk behaviours as a result of the face-to-face interview, failure to explore or define the effects of separation from families and cultural factors in predicting risk behaviours of elderly people, and the inability to determine CD4 counts and viral loads all limit the interpretation and generalizability of the data. Further studies need to be done to verify the results presented herein and also determine the exact prevalence of HIV among the elderly in the country.

## Conclusion

In conclusion, the prevalence of HIV (4.18 %) among the elderly people on admission at both SME and Medical Wards at the KBTH is higher than the national prevalence (1.37 %). National HIV prevalence data for elderly Ghanaians is not currently available but should be collected. Based on these data, there should be considerations to include the elderly in HIV/AIDS prevention and control programmes that provide general HIV and STI education.

## Abbreviations

AIDS: Acquired Immune Deficiency Syndrome
HIV: The human immunodeficiency virus
KBTH: Korle Bu Teaching Hospital
NACP: National AIDS Control Programme
SME: Surgical/Medical Emergency
UGSMD: University of Ghana School of Medicine and Dentistry
